# Supplementary material for: Social network characteristics associated with mid-to-older aged adults’ co-engagement in physical activity
Source: PLoS One. 2025 May 7;20(5):e0319981. doi: 10.1371/journal.pone.0319981 (PMC12057854; doi:10.1371/journal.pone.0319981)
Supplement: S1 Appendix — (PDF) [file pone.0319981.s001.pdf]

Updated_PACES_FINAL.netcanvas

LAST MODIFIED:10/3/2022, 11:26:55 AM

SCHEMA VERSION:7

DOCUMENT CREATED: 3/31/2024 12:17:30 PM

Contents

STAGES

1. Intro
2. Ego data
3. Physical activity (IPAQ)
4. Vigorous physical activity (days)
5. Vigorous Physical Activity (time)
6. Moderate physical activity (days)
7. Moderate physical activity (time)
8. Physical activity - walking
9. Walking (time)
10. Strength and balance
11. Sitting
12. Health and Wellbeing
13. Recent mood
14. Community environment
15. Introduction to Social Networks
16. Name generator 1
17. Name generator 2
18. Name generator 3
19. Name generator 4
20. Alter attributes
21. Alter attributes - relationship to ego
22. Alter attributes - gender
23. Alter attributes - age
24. Alter attributes - relationship length
25. Alter attributes - health
26. Per alter form
27. Alter attributes - ease
28. Alter attributes - counting on
29. Closeness and alter-alter ties
30. Name generator 5 - Groups
31. Group attributes
32. Places stage
33. Name generator - Places
34. Place attributes
35. Debrief Form

CODEBOOK

Ego

NODE TYPES

Person

Group

Place

EDGE TYPES

Know_each_other

Activities_with

ASSETS

IMAGE

63127038-62273800-bf7f-11e9-914f-bd1c431c76f2.png

Debrief.png

NETWORK

Places V9.csv

1 Intro

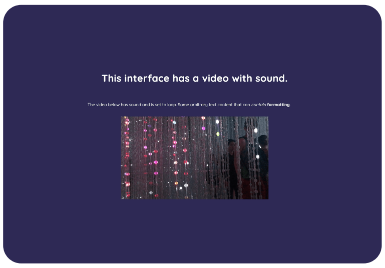

PAGE HEADING

Thank you for agreeing to take part in the Social Network survey portion of the PACES study.

ITEMS

NAME	63127038-62273800-bf7f-11e9-914f-bd1c431c76f2.png
BLOCK SIZE	SMALL
TYPE	Image
PREVIEW	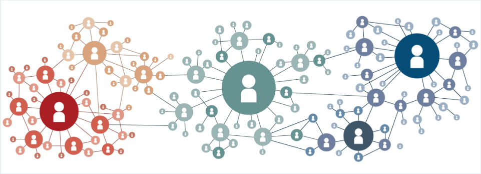

INTERVIEWER SCRIPT

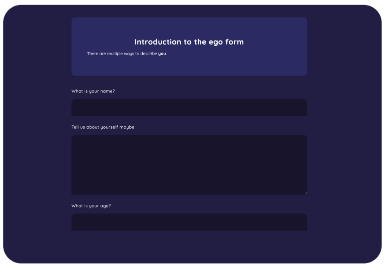

2

Ego data

INTRODUCTION PANEL

About You

First, we would like to know a little bit about you. Please answer the following questions:

FORM

VARIABLE	COMPONENT	PROMPT
DOB	DatePicker	1. What is your date of birth?
Location_Single	RadioGroup	2. Do you live in:
Postcode	Text	3. What is your postcode? (where you live most of the time)
Residency	RadioGroup	4. How long have you lived in the town/ city where you live now?
Education	RadioGroup	5. What is your highest level of schooling?
Employment_status	RadioGroup	6a. Which of the following best describes your current employment situation?
Work_from_home	RadioGroup	6b. If you are employed, have you changed to work-from-home due to Covid?
Days_work_from_home	Number	6c. If yes, how many days per week do you work from home?
Finances	RadioGroup	7. How well would you say you are managing financially these days?

VARIABLE	COMPONENT	PROMPT
Gender	RadioGroup	8a. How would you describe your gender?
Gender_other	TextArea	8b. If you do not identify as a man or a woman, please write in what is best:
relationship_status	RadioGroup	9. What is your relationship status?
Sex_orient	RadioGroup	10. What is your sexual orientation?
Ethnicity	RadioGroup	11. What is your ethnic group?
Frailty_a	RadioGroup	12 a. In the last year, have you lost more than 4.5 kg unintentionally? (i.e. not due to dieting or exercise)
Frailty_b	RadioGroup	12b. How often did you feel that everything you did was an effort?
Frailty_c	RadioGroup	12c. Can you reach the other side of the road when the light turns green at a pedestrian crossing?
Frailty_d	RadioGroup	12d. Do you experience difficulties in daily life because of low grip strength?
Transitions_multi	CheckboxGroup	13. Have you experienced any of the following events in the last 2-5 years? Mark all that apply.
Dog_owner	RadioGroup	14. Do you own a dog?

INTERVIEWER SCRIPT

3 Physical activity (IPAQ)

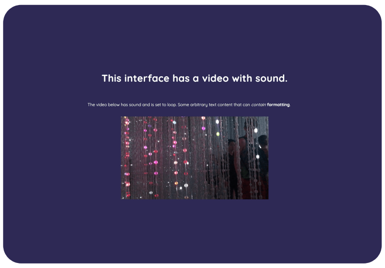

PAGE HEADING

Physical activity (IPAQ)

ITEMS

BLOCK SIZE	MEDIUM
TYPE	Text
CONTENT	We are interested in finding out about the kinds of physical activities that people do as part of their everyday lives. The questions will ask you about the time you spent being physically active in the last 7 days. Please answer each question even if you do not consider yourself to be an active person. Please think about the activities you do at work, as part of your house and garden/yard work, to get from place to place, and in your spare time for recreation, exercise or sport.

INTERVIEWER SCRIPT

4 Vigorous physical activity (days)

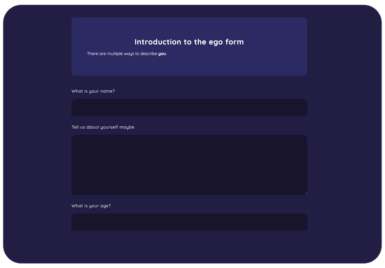

INTRODUCTION PANEL

Vigorous physical activity

Think about all the **vigorous** activities that you did in the **last 7 days**. **Vigorous** physical activities refer to activities that take hard physical effort and make you breathe much harder than normal. Think *only* about those physical activities that you did for at least 10 minutes at a time.

FORM

VARIABLE	COMPONENT	PROMPT
123 Vig_activity_days	Number	1. During the last 7 days, on how many days did you do vigorous physical activities like heavy lifting, digging, aerobics, or fast bicycling?

INTERVIEWER SCRIPT

5 Vigorous Physical Activity (time)

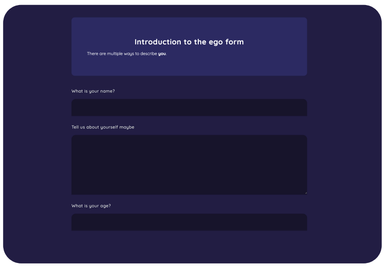

SKIP LOGIC

ACTION	SHOW
RULES	Ego has 123 Vig_activity_days that is greater than 0

INTRODUCTION PANEL

Vigorous activity

We would like to know how much time you spend doing vigorous physical activity

FORM

VARIABLE	COMPONENT	PROMPT
123 Vig_minutes	Number	2. How much time **(in minutes)** did you usually spend doing **vigorous** physical activities on one of those days?

INTERVIEWER SCRIPT

6 Moderate physical activity (days)

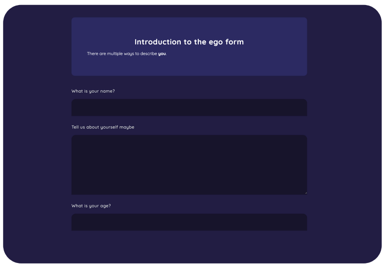

INTRODUCTION PANEL

Moderate physical activity

Think about all the moderate activities that you did in the last 7 days. Moderate activities refer to activities that take moderate physical effort and make you breathe somewhat harder than normal. Think only about those physical activities that you did for at least 10 minutes at a time.

FORM

VARIABLE	COMPONENT	PROMPT
123 Mod_days	Number	3. During the last 7 days, on how many days did you do moderate physical activities like carrying light loads, bicycling at a regular pace, or doubles tennis? Do not include walking.

INTERVIEWER SCRIPT

7 Moderate physical activity (time)

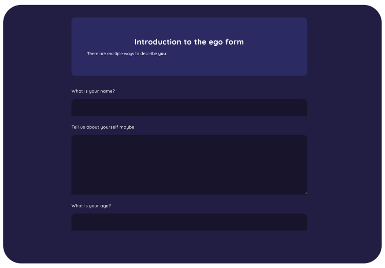

SKIP LOGIC

ACTION	SHOW
RULES	Ego has 123 Mod_days that is greater than 0

INTRODUCTION PANEL

We would like to know how much time you spend doing moderate physical activity

4. How much time **(in minutes)** did you usually spend doing moderate physical activities on one of those days?

FORM

VARIABLE	COMPONENT	PROMPT
123 Mod_minutes	Number	4. How much time (in minutes) did you usually spend doing moderate physical activities on one of those days?

INTERVIEWER SCRIPT

8 Physical activity - walking

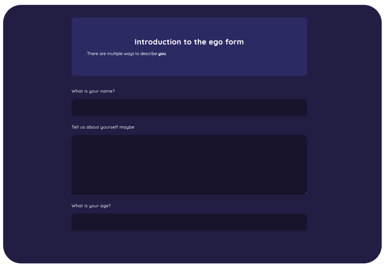

INTRODUCTION PANEL

Walking

Think about the time you spent walking in the last 7 days. This includes at work and at home, walking to travel from place to place, and any other walking that you have done solely for recreation, sport, exercise, or leisure.

FORM

VARIABLE	COMPONENT	PROMPT
123 Walk_days	Number	5. During the last 7 days, on how many days did you walk for at least 10 minutes at a time?

INTERVIEWER SCRIPT

9 Walking (time)

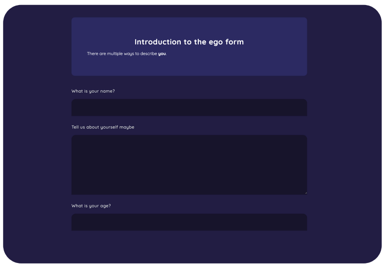

SKIP LOGIC

ACTION	SHOW
RULES	Ego has 123 Walk_days that is greater than 0

INTRODUCTION PANEL

Walking

We would like to know how much time you spend walking

FORM

VARIABLE	COMPONENT	PROMPT
123 Walk_minutes	Number	6. How much time **in minutes** did you usually spend walking on one of those days?

INTERVIEWER SCRIPT

10 Strength and balance

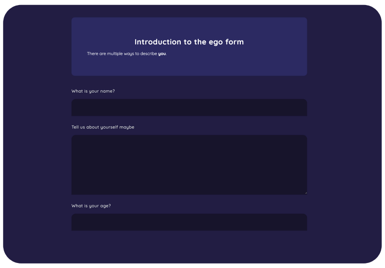

INTRODUCTION PANEL

Strength and balance

The next section is about muscle strengthening and balance improvement activities that you have done in the last 7 days. This may include sport and exercise activities, or alternatively domestic activities such as carrying shopping, manual labouring or heavy gardening tasks.

FORM

VARIABLE	COMPONENT	PROMPT
123 Strength_activity	Number	7. During the last 7 days, on how many days did you do any of the physical activities mentioned above, or undertake any other physical activities where the effort was usually enough to make your muscles feel some tension, shake or feel warm?
123 Balance_act	Number	8. During the last 7 days, on how many days did you do activities to improve your balance? For example, activities that included standing on one leg or rising on to tip-toes, with or without support, walking sideways by crossing one foot over the other, or by placing one heel directly in front of the other foot's toes.

INTERVIEWER SCRIPT

11 Sitting

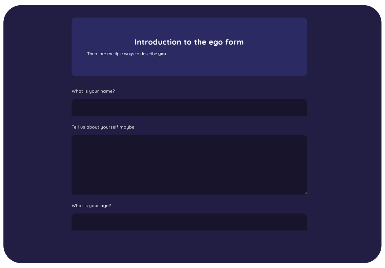

INTRODUCTION PANEL

Time spent sitting

The last question is about the time you spent sitting on weekdays during the last 7 days. Include time spent at work, at home, while doing course work and during leisure time. This may include time spent sitting at a desk, visiting friends, reading, or sitting or lying down to watch television.

FORM

VARIABLE	COMPONENT	PROMPT
123 Sitting_time	Number	9. During the last 7 days, how much time **in minutes** did you spend sitting on a weekday?

INTERVIEWER SCRIPT

12 Health and Wellbeing

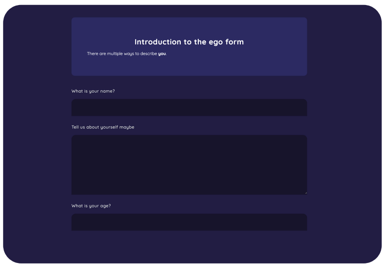

INTRODUCTION PANEL

Health and Wellbeing

In the following section, we would like to ask about your recent experiences of health and wellbeing, including companionship.

FORM

VARIABLE	COMPONENT	PROMPT
UCLA_3a	RadioGroup	10a. How often do you feel that you have no one to talk to?
UCLA_3b	RadioGroup	10b. How often do you feel left out?
UCLA_3c	RadioGroup	10c. How often do you feel alone?
Lonely_direct	RadioGroup	11. How often do you feel lonely?
EQ-5D_MOB	RadioGroup	12a. Please tick the ONE box that best describes your health TODAY. (MOBILITY)
EQ-5D_SC	RadioGroup	12b. Please tick the ONE box that best describes your health TODAY (SELF-CARE)
EQ-5D_Activ	RadioGroup	12c. Please tick the ONE box that best describes your health TODAY. (USUAL ACTIVITIES e.g. work, study, housework, family or leisure activities).
EQ-5D_Pain	RadioGroup	12d. Please tick the ONE box that best describes your health TODAY. (PAIN AND DISCOMFORT)
EQ-5D_ANXDEP	RadioGroup	12e. Please tick the ONE box that best describes your health TODAY. (ANXIETY AND DEPRESSION)

INTERVIEWER SCRIPT

13 Recent mood

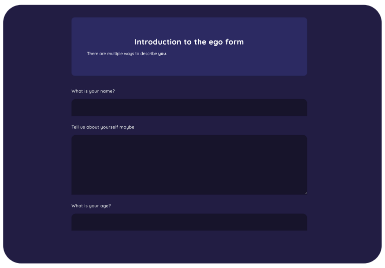

INTRODUCTION PANEL

Recent mood

In this section, we would like to learn about your mood in the past two weeks.

13. Over the last 2 weeks, how often have you been bothered by the following problems?

FORM

VARIABLE	COMPONENT	PROMPT
PHQ4_1	RadioGroup	13a. Feeling nervous, anxious or on edge
PHQ4_2	RadioGroup	13b. Not being able to stop or control worrying
PHQ4_3	RadioGroup	13c. Having little interest or pleasure in doing things
PHQ4_4	RadioGroup	13d. Feeling down, depressed or hopeless

INTERVIEWER SCRIPT

14 Community environment

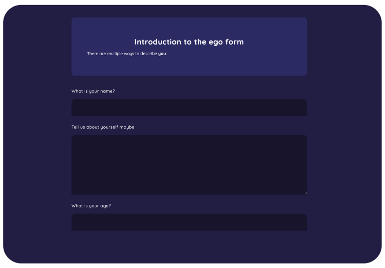

INTRODUCTION PANEL

Community environment

Now we would like to know how you feel about your local community.

FORM

VARIABLE	COMPONENT	PROMPT
Trust	LikertScale	14. People in this neighbourhood can be trusted.
Similar	LikertScale	15. I am similar to others in my neighbourhood.
Access	RadioGroup	16. Are you able to access all services such as healthcare, food shops or learning facilities within a 20 minute journey from your house?
Chat	RadioGroup	17. How often do you chat to any of your neighbours, more than to just say hello?

INTERVIEWER SCRIPT

15 Introduction to Social Networks

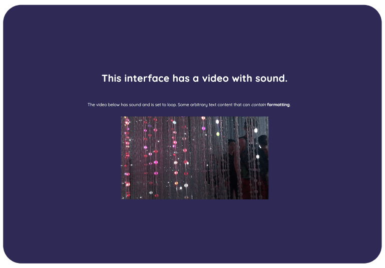

PAGE HEADING

Your Social Networks

ITEMS

BLOCK SIZE	LARGE
TYPE	Text
CONTENT	**Now I will ask you a series of questions about the people in your life.**<br><br>To start, please write the names of anyone whom you have spent time with or socialised with in the last month. It can be helpful to think about the people you have talked to for more than 5 minutes (more than just saying hello). This can be face-to-face for instance, for example going to the park, out for drinks or a cuppa, exercising, or just to catch-up. It can also be remotely, such as by texting, phone calls, or via social media.<br><br>You can also think about the people that you may share good, or bad, or interesting news with or whose advice you would seek when making decisions about important matters.<br><br>For example, you can include individuals who are friends, family, neighbours, colleagues, or even someone you chat with at the shops or around town.<br><br>Remember, all your answers are confidential, and all the names you give will be replaced with ID numbers. We will not show anyone else what names you type, and we will delete all names for analysis. We only use these names to help you answer later questions (e.g. 'How old is John?')

INTERVIEWER SCRIPT

16 Name generator 1

SUBJECT	Person
VARIABLES	Name

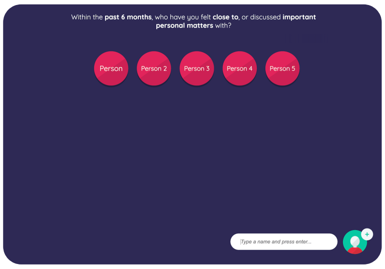

QUICK ADD

VARIABLE	Name
TYPE	text

PROMPTS

1. 1. To start, please write the names of anyone whom you have spent time with or socialised with in the last month.

INTERVIEWER SCRIPT

It can be helpful to think about the people you have talked to for more than 5 minutes (more than just saying hello). This can be face-to-face for instance, for example going to the park, out for drinks or a cuppa, exercising, or just to catch-up. It can also be remotely, such as by texting, phone calls, or via social media. You can also think about the people that you may share good, or bad, or interesting news with or whose advice you would seek when making decisions about important matters.

For example, you can include individuals who are friends, family, neighbours, colleagues, or even someone you chat with at the shops or around town. Remember, all your answers are confidential, and all the names you give will be replaced with ID numbers. We will not show anyone else what names you type, and we will delete all names for analysis. We only use these names to help you answer later questions (e.g. 'How old is John?')

17 Name generator 2

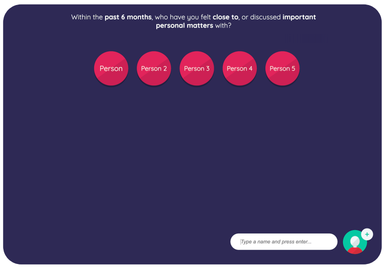

SUBJECT	Person
VARIABLES	Name

QUICK ADD

VARIABLE	Name
TYPE	text

PROMPTS

1. 2. Are there people whom you feel connected to, but you have not seen or spoken to in the last month? E.g. people you don't speak to often, but you still consider yourself connected.

INTERVIEWER SCRIPT

Explain that this can be a friend/family member who doesn't locally and you don't speak often, but you still consider yourself connected.

18 Name generator 3

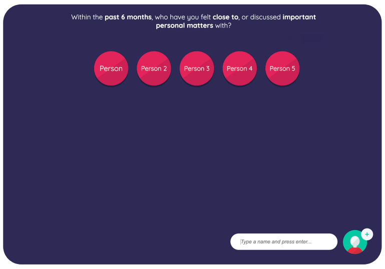

SUBJECT	Person
VARIABLES	Activities_with, Name

QUICK ADD

VARIABLE	Name
TYPE	text

PANELS

1.

TITLE	People you have mentioned
DATA SOURCE	*Existing network*

PROMPTS

1. 3. Are there people whom you do physical activities with, like going for a walk, playing sports, going to an exercise class, or gardening?

VARIABLE	VALUE
Activities_with	*TRUE*

INTERVIEWER SCRIPT

19 Name generator 4

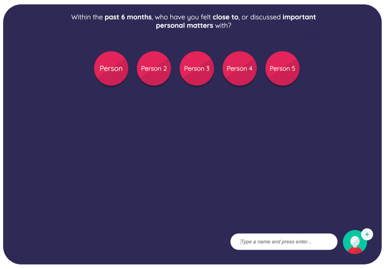

SUBJECT	Person
VARIABLES	Name

QUICK ADD

VARIABLE	Name
TYPE	text

PROMPTS

1. 4. Is there anyone else whom you frequently chat with, but you don’t necessarily know well or don’t know their name? E.g. the postman or someone who delivers groceries to your house?

INTERVIEWER SCRIPT

It is not necessary to label these people by name. Labels like 'postman', 'delivery person', etc. are fine.

20 Alter attributes

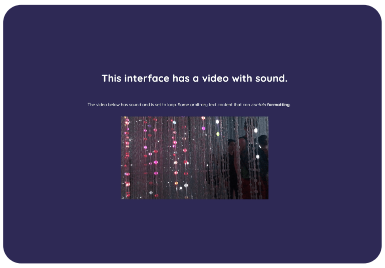

PAGE HEADING

About the people in your network

ITEMS

BLOCK SIZE	SMALL
TYPE	Text
CONTENT	We would like to get to know the people you have mentioned above a bit better. The next screen will ask you questions about each person.<br><br>Remember, all of this information is confidential. We will remove names and replace them with ID numbers for analysis.

INTERVIEWER SCRIPT

21 Alter attributes - relationship to ego

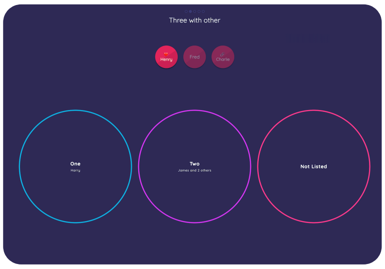

SUBJECT	Person
VARIABLES	Relat

PROMPTS

1. 1. What is this person's relationship to you?

VARIABLE	Relat

INTERVIEWER SCRIPT

22 Alter attributes - gender

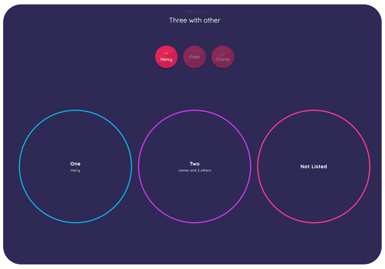

SUBJECT	Person
VARIABLES	Alter_gender

PROMPTS

1. 2. What is their gender?

VARIABLE	Alter_gender

INTERVIEWER SCRIPT

23 Alter attributes - age

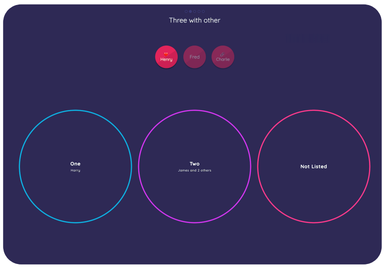

SUBJECT	Person
VARIABLES	Alter_age

PROMPTS

1. 3. What is their age?

VARIABLE	Alter_age

INTERVIEWER SCRIPT

24 Alter attributes - relationship length

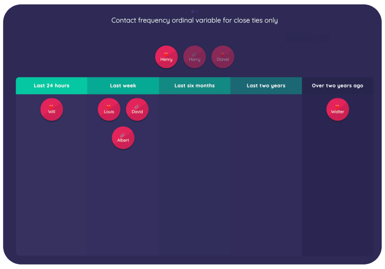

SUBJECT	Person
VARIABLES	Rel_length

PROMPTS

1. 4. How long have you known them?

VARIABLE	Rel_length

INTERVIEWER SCRIPT

25 Alter attributes - health

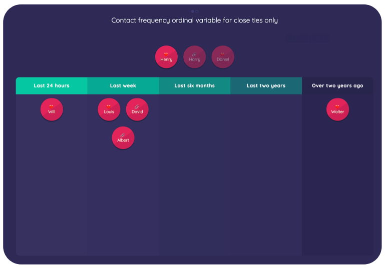

SUBJECT	Person
VARIABLES	Alter_health

PROMPTS

1. 5. How would you rate their physical health?

VARIABLE	Alter_health

INTERVIEWER SCRIPT

26 Per alter form

SUBJECT	Person
VARIABLES	Area, City, Closeness, Freq, Live_close, Postcode

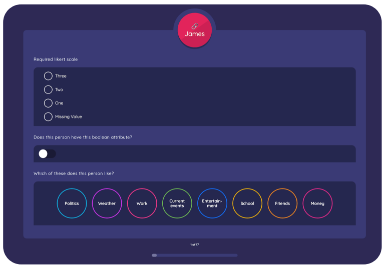

INTRODUCTION PANEL

More about the people in your network

Thinking about each person you have nominated, please answer the following questions

FORM

VARIABLE	COMPONENT	PROMPT
Closeness	LikertScale	6. On a scale of 1 to 5, where 5 is very close and 1 is not very close, how close do you feel to this person?
Live_close	RadioGroup	7. How close do you live to this person?
City	Text	8. Which city do they live in? If they live abroad, write in the country too.
Area	Text	9. Which neighbourhood do they live in?
Postcode	Text	10. Write in their postcode (if known).
Freq	RadioGroup	11. How often do you interact with them? This could be in person, over the phone, or via social media, such as texting, WhatsApp, or the internet.

INTERVIEWER SCRIPT

27 Alter attributes - ease

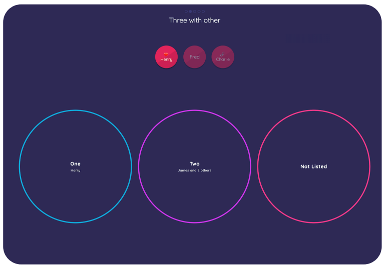

SUBJECT	Person
VARIABLES	Ease_with

PROMPTS

1. 12. Below is a list of all the people you have nominated. Please select those who fit each of the criteria

VARIABLE	Ease_with

INTERVIEWER SCRIPT

28 Alter attributes - counting on

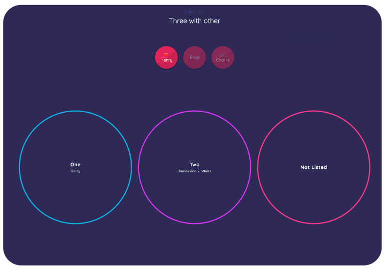

SUBJECT	Person
VARIABLES	Count_on

PROMPTS

1. 13. Below is a list of all the people you nominated above. Please select those who fit the criteria

VARIABLE	Count_on

INTERVIEWER SCRIPT

29 Closeness and alter-alter ties

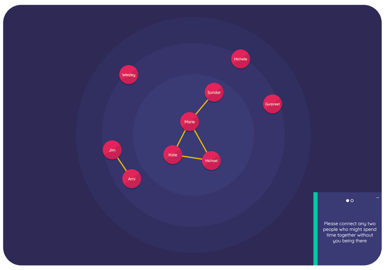

SUBJECT	Person
VARIABLES	Knows

PROMPTS

1. 14. This screen shows all the people you have mentioned so far. Position the people you feel close to near the centre and those who you feel less close to further out.

LAYOUT VARIABLE	Knows

2. 15. Thinking about the people you have mentioned today – who could they have a conversation with, without you being present? Click on one and then the other person to connect them.

LAYOUT VARIABLE	Knows
CREATES EDGE	Know_each_other
ALLOW HIGHLIGHTING	*FALSE*

INTERVIEWER SCRIPT

We would now like to know how well people in your social network know each other. For each individual you have named, please select the other people (from your social network) whom they could spent time with or have a conversation with without you being present.

30 Name generator 5 - Groups

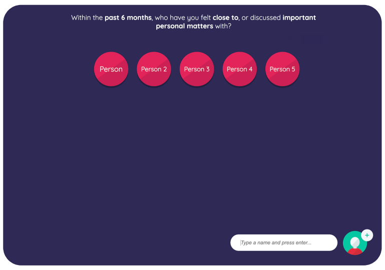

SUBJECT	Group
VARIABLES	Group, Name

QUICK ADD

VARIABLE	Name
TYPE	text

PROMPTS

1. 16. Are there community groups, clubs, groups, or teams that you go to?

VARIABLE	VALUE
Group	*TRUE*

INTERVIEWER SCRIPT

31 Group attributes

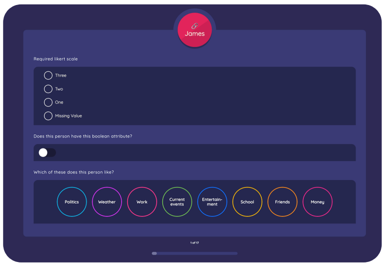

SUBJECT	Group
VARIABLES	Group_age, Group_gender, Group_location, Group_size, Group_type

NETWORK FILTERING

RULES	Group exists

INTRODUCTION PANEL

Group attributes

Thinking about each of the groups you have mentioned, answer the following questions.

FORM

VARIABLE	COMPONENT	PROMPT
Group_type	Text	17. What type of group is it / what activities do you do?
Group_location	Text	18. Where is it? (Write in the city, neighbourhood, postcode)
Group_size	Number	19. How many people regularly attend with you?
Group_age	RadioGroup	20. In terms of age, the group is mostly:
Group_gender	RadioGroup	21. In terms of gender, the group is mostly:

INTERVIEWER SCRIPT

32 Places stage

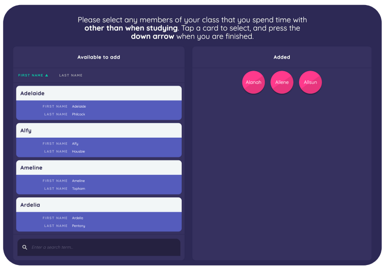

DATASOURCE

TYPE	network
NAME	Places V9.csv

PROMPTS

1. 1. We would now like to know the places in your community where you go to socialise or to get some physical activity. Please tell us the name of the places, and we will help find them.

INTERVIEWER SCRIPT

When we say community, we mean the general area of your local authority.

33 Name generator - Places

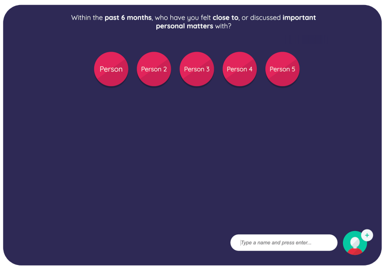

SUBJECT	Place
VARIABLES	Name

QUICK ADD

VARIABLE	Name
TYPE	text

PROMPTS

1. 2. Where else in your community do you go to socialise or to get some physical activity? You can now add places that were not listed within the previous question.

INTERVIEWER SCRIPT

34 Place attributes

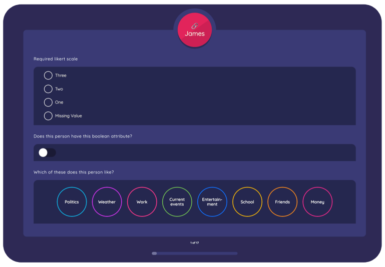

SUBJECT	Place
VARIABLES	Active_there, Affect_activity, Affect_social, Easy_get_there, Get_there, Meet_there

NETWORK FILTERING

RULES	Place exists

INTRODUCTION PANEL

Places characteristics

Finally, we would like to ask you some questions about each of these places.

FORM

VARIABLE	COMPONENT	PROMPT
Get_there	RadioGroup	1. Do you walk or drive or take public transport to get there?
Easy_get_there	LikertScale	2. How easy is it to get there?
Meet_there	RadioGroup	3. Do you meet your friends or family there?
Active_there	RadioGroup	4. Are you physically active when there?
Affect_activity	LikertScale	If this placed closed, how much would it affect your physical activity?
Affect_social	LikertScale	5. If this place closed, how much would it affect your social life?

INTERVIEWER SCRIPT

35 Debrief Form

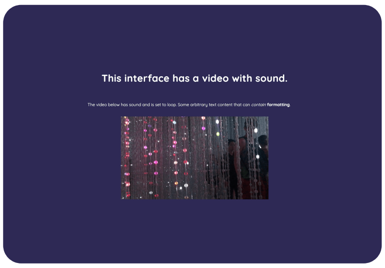

PAGE HEADING

Debrief Form

ITEMS

NAME	Debrief.png
BLOCK SIZE	LARGE
TYPE	Image
PREVIEW	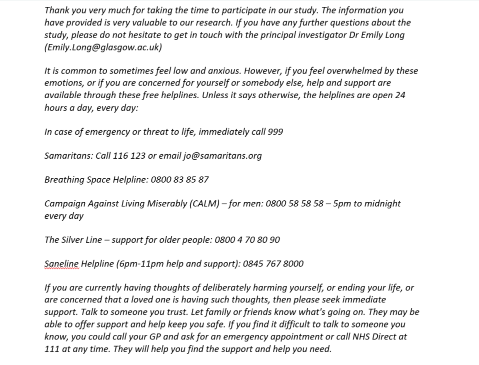

Thank you very much for taking the time to participate in our study. The information you have provided is very valuable to our research. If you have any further questions about the study, please do not hesitate to get in touch with the principal investigator Dr Emily Long (Emily.Long@glasgow.ac.uk)

It is common to sometimes feel low and anxious. However, if you feel overwhelmed by these emotions, or if you are concerned for yourself or somebody else, help and support are available through these free helplines. Unless it says otherwise, the helplines are open 24 hours a day, every day:

In case of emergency or threat to life, immediately call 999

Samaritans: Call 116 123 or email jo@samaritans.org

Breathing Space Helpline: 0800 83 85 87

Campaign Against Living Miserably (CALM) – for men: 0800 58 58 58 – 5pm to midnight every day

The Silver Line – support for older people: 0800 4 70 80 90

Saneline Helpline (6pm-11pm help and support): 0845 767 8000

If you are currently having thoughts of deliberately harming yourself, or ending your life, or are concerned that a loved one is having such thoughts, then please seek immediate support. Talk to someone you trust. Let family or friends know what's going on. They may be able to offer support and help keep you safe. If you find it difficult to talk to someone you know, you could call your GP and ask for an emergency appointment or call NHS Direct at 111 at any time. They will help you find the support and help you need.

INTERVIEWER SCRIPT

Ego

Name	Type	Used In
Access	ordinal<br>VALUE / LABEL<br>1 / Yes<br>2 / No	Community environment
Balance_act	number	Strength and balance
Chat	ordinal<br>VALUE / LABEL<br>5 / On most days<br>4 / Once or twice a week<br>3 / Less than once a month<br>2 / Never<br>1 / Don't have any neighours	Community environment
Days_work_from_home	number	Ego data
DOB	datetime	Ego data
Dog_owner	ordinal<br>VALUE / LABEL<br>1 / Yes<br>2 / No	Ego data

Name	Type	Used In
Education	ordinal	Ego data

VALUE	LABEL
1	Completed primary school or less
2	Secondary school, not completed
3	Completed secondary school
4	Completed college or university
5	Postgraduate qualification, not completed (MA or MSc or PhD or similar)
6	Completed postgraduate qualification (MA or MSc or PhD or similar)
7	I don't know

Name	Type	Used In
Employment_status	ordinal	Ego data

VALUE	LABEL
1	In paid employment (full or part time)
2	Unemployed
3	Retired
4	Long-term sick or disabled

<table>
<tr><th>Name</th><th>Type</th><th>Used In</th></tr>
<tr><td>EQ-5D_Activ</td><td>ordinal
<table>
<tr><th>VALUE</th><th>LABEL</th></tr>
<tr><td>5</td><td>I have no problems doing my usual activities</td></tr>
<tr><td>4</td><td>I have slight problems doing my usual activities</td></tr>
<tr><td>3</td><td>I have moderate problems doing my usual activities</td></tr>
<tr><td>2</td><td>I have severe problems doing my usual activities</td></tr>
<tr><td>1</td><td>I am unable to do my usual activities</td></tr>
</table></td><td>Health and Wellbeing</td></tr>
<tr><td>EQ-5D_ANXDEP</td><td>ordinal
<table>
<tr><th>VALUE</th><th>LABEL</th></tr>
<tr><td>5</td><td>I am not anxious or depressed</td></tr>
<tr><td>4</td><td>I am slightly anxious or depressed</td></tr>
<tr><td>3</td><td>I am moderately anxious or depressed</td></tr>
<tr><td>2</td><td>I am severely anxious or depressed</td></tr>
<tr><td>1</td><td>I am extremely anxious or depressed</td></tr>
</table></td><td>Health and Wellbeing</td></tr>
</table>

Name	Type	Used In
EQ-5D_MOB	ordinal	Health and Wellbeing

VALUE	LABEL
5	I have no problems in walking about
4	I have slight problems in walking about
3	I have moderate problems in walking about
2	I have severe problems in walking about
1	I am unable to walk about

Name	Type	Used In
EQ-5D_Pain	ordinal	Health and Wellbeing

VALUE	LABEL
5	I have no pain or discomfort
4	I have slight pain or discomfort
3	I have moderate pain or discomfort
2	I have severe pain or discomfort
1	I have extreme pain or discomfort

<table>
<tr><th>Name</th><th>Type</th><th>Used In</th></tr>
<tr><td>EQ-5D_SC</td><td>ordinal
<table>
<tr><th>VALUE</th><th>LABEL</th></tr>
<tr><td>5</td><td>I have no problems washing or dressing myself</td></tr>
<tr><td>4</td><td>I have slight problems washing or dressing myself</td></tr>
<tr><td>3</td><td>I have moderate problems washing or dressing myself</td></tr>
<tr><td>2</td><td>I have severe problems washing or dressing myself</td></tr>
<tr><td>1</td><td>I am unable to wash or dress myself</td></tr>
</table></td><td>Health and Wellbeing</td></tr>
<tr><td>Ethnicity</td><td>ordinal</td><td>Ego data</td></tr>
</table>

Name	Type	Used In

VALUE	LABEL
1	White British/English/Scottish/Welsh/Northern Irish/Irish
2	Any other White background
3	White and Black Caribbean
4	White and Black African
5	White and Asian
6	Any other mixed background
7	Indian
8	Pakistani
9	Bangladeshi
10	Chinese
11	Any other Asian background
12	Caribbean
13	African
14	Any other Black background
15	Latin American
16	Any other ethnic group

Name	Type	Used In
Finances	ordinal	Ego data

VALUE	LABEL
1	Living comfortably
2	Doing alright
3	Just getting by
4	Finding it quite difficult
5	Finding it very difficult

Name	Type	Used In
Frailty_a	ordinal	Ego data

VALUE	LABEL
1	Yes
2	No

Name	Type	Used In
Frailty_b	ordinal<br><br>VALUE / LABEL<br>1 Always<br>2 Most of the time<br>3 Sometimes<br>4 Occasionally<br>5 Never	Ego data
Frailty_c	ordinal<br><br>VALUE / LABEL<br>1 Yes, without any trouble<br>2 Yes, with difficulty<br>3 No	Ego data
Frailty_d	ordinal<br><br>VALUE / LABEL<br>1 Yes<br>2 No	Ego data
Gender	ordinal<br><br>VALUE / LABEL<br>1 Man<br>2 Woman<br>3 Other	Ego data
Gender_other	text	Ego data
Location	categorical<br><br>VALUE / LABEL<br>1 Renfrewshire<br>2 South Lanarkshire	

<table>
<tr><th>Name</th><th>Type</th><th>Used In</th></tr>
<tr><td>Location_Single</td><td>ordinal<table><tr><th>VALUE</th><th>LABEL</th></tr><tr><td>1</td><td>Renfrewshire</td></tr><tr><td>2</td><td>South Lanarkshire</td></tr></table></td><td>Ego data</td></tr>
<tr><td>Lonely_direct</td><td>ordinal<table><tr><th>VALUE</th><th>LABEL</th></tr><tr><td>5</td><td>Often or always</td></tr><tr><td>4</td><td>Some of the time</td></tr><tr><td>3</td><td>Occasionally</td></tr><tr><td>2</td><td>Hardly ever</td></tr><tr><td>1</td><td>Never</td></tr></table></td><td>Health and Wellbeing</td></tr>
<tr><td>Mod_days</td><td>number</td><td>Moderate physical activity (days)
Moderate physical activity (time)</td></tr>
<tr><td>Mod_minutes</td><td>number</td><td>Moderate physical activity (time)</td></tr>
<tr><td>PHQ4_1</td><td>ordinal<table><tr><th>VALUE</th><th>LABEL</th></tr><tr><td>0</td><td>Not at all</td></tr><tr><td>1</td><td>Several days</td></tr><tr><td>2</td><td>More than half the days</td></tr><tr><td>3</td><td>Nearly every day</td></tr></table></td><td>Recent mood</td></tr>
<tr><td>PHQ4_2</td><td>ordinal<table><tr><th>VALUE</th><th>LABEL</th></tr><tr><td>0</td><td>Not at all</td></tr><tr><td>1</td><td>Several days</td></tr><tr><td>2</td><td>More than half the days</td></tr><tr><td>3</td><td>Nearly every day</td></tr></table></td><td>Recent mood</td></tr>
</table>

Name	Type	Used In
PHQ4_3	ordinal	Recent mood

VALUE	LABEL
0	Not at all
1	Several days
2	More than half the days
3	Nearly every day

Name	Type	Used In
PHQ4_4	ordinal	Recent mood

VALUE	LABEL
0	Not at all
1	Several days
2	More than half the days
3	Nearly every day

Name	Type	Used In
Postcode	text	Ego data
relationship_status	ordinal	Ego data

VALUE	LABEL
1	Single and never married or never in a legally recognised Civil Partnership
2	Married
3	A Civil Partner in a legally recognised Civil Partnership
4	Partner/girlfriend/boyfriend
5	Separated but legally married
6	Divorced
7	Widowed

Name	Type	Used In
Residency	ordinal	Ego data

VALUE	LABEL
1	Less than a year
2	1-2 years
3	3-5 years
4	6-10 years
5	More than 10 years

Name	Type	Used In
Sex_orient	ordinal	Ego data

VALUE	LABEL
1	Heterosexual or straight
2	Gay or lesbian
3	Bisexual
4	Other
5	Prefer not to say

Name	Type	Used In
Similar	ordinal	Community environment

VALUE	LABEL
5	Strongly agree
4	Agree
3	Neither agree nor disagree
2	Disagree
1	Strongly disagree

Name	Type	Used In
123 Sitting_time	number	Sitting
123 Strength_activity	number	Strength and balance

<table>
<tr><th>Name</th><th>Type</th><th>Used In</th></tr>
<tr><td>Transitions</td><td>ordinal<table>
<tr><th>VALUE</th><th>LABEL</th></tr>
<tr><td>1</td><td>Retired</td></tr>
<tr><td>2</td><td>Diagnosed with a health condition</td></tr>
<tr><td>3</td><td>Moved to a new area</td></tr>
<tr><td>4</td><td>Separated/Divorced</td></tr>
<tr><td>5</td><td>Bereavement</td></tr>
<tr><td>6</td><td>Became a carer</td></tr>
<tr><td>7</td><td>None of the above</td></tr>
</table></td><td></td></tr>
<tr><td>Transitions_multi</td><td>categorical<table>
<tr><th>VALUE</th><th>LABEL</th></tr>
<tr><td>1</td><td>Retired</td></tr>
<tr><td>2</td><td>Diagnosed with a health condition</td></tr>
<tr><td>3</td><td>Moved to a new area</td></tr>
<tr><td>4</td><td>Divorced/separated</td></tr>
<tr><td>5</td><td>Bereavement</td></tr>
<tr><td>6</td><td>Became a carer</td></tr>
<tr><td>7</td><td>None of the above</td></tr>
</table></td><td>Ego data</td></tr>
<tr><td>Trust</td><td>ordinal<table>
<tr><th>VALUE</th><th>LABEL</th></tr>
<tr><td>1</td><td>Strongly disagree</td></tr>
<tr><td>2</td><td>Disagree</td></tr>
<tr><td>3</td><td>Neither agree nor disagree</td></tr>
<tr><td>4</td><td>Agree</td></tr>
<tr><td>5</td><td>Strongly agree</td></tr>
</table></td><td>Community environment</td></tr>
</table>

Name	Type	Used In
UCLA_3a	ordinal<br>VALUE / LABEL<br>1 / Hardly ever or never<br>2 / Some of the time<br>3 / Often	Health and Wellbeing
UCLA_3b	ordinal<br>VALUE / LABEL<br>1 / Hardly ever or never<br>2 / Some of the time<br>3 / Often	Health and Wellbeing
UCLA_3c	ordinal<br>VALUE / LABEL<br>1 / Hardly ever or never<br>2 / Some of the time<br>3 / Often	Health and Wellbeing
Vig_activity_days	number	Vigorous physical activity (days)<br>Vigorous Physical Activity (time)
Vig_minutes	number	Vigorous Physical Activity (time)
Walk_days	number	Physical activity - walking<br>Walking (time)
Walk_minutes	number	Walking (time)

Name	Type	Used In
Work_from_home	ordinal	Ego data

VALUE	LABEL
1	Yes.
2	No.
3	I am not currently employed.

Person

Name	Type	Used In
Activities_with	boolean	Name generator 3
Alter_age	categorical	Alter attributes - age
Alter_gender	categorical	Alter attributes - gender
Alter_health	ordinal	Alter attributes - health

Alter_age (categorical):

VALUE	LABEL
1	Less than 20 years old
2	20-30
3	31-40
4	41-50
5	51-60
6	61-70
7	71-80
8	Older than 80

Alter_gender (categorical):

VALUE	LABEL
1	Male
2	Female
3	Other

Alter_health (ordinal):

VALUE	LABEL
1	Very poor
2	Poor
3	Neither good nor poor
4	Good
5	Very good
6	I don't know

Name	Type	Used In
Area	text	Per alter form
City	text	Per alter form
Closeness	ordinal<br><br>VALUE / LABEL<br>1 / 1<br>2 / 2<br>3 / 3<br>4 / 4<br>5 / 5	Per alter form
Count_on	categorical<br><br>VALUE / LABEL<br>1 / I feel close to this person and could call on them in times of need.<br>2 / I don't feel this way about them.<br>3 / I don't know.	Alter attributes - counting on
Ease_with	categorical<br><br>VALUE / LABEL<br>1 / I feel at ease with this person and can talk to them about personal and sensitive matters<br>2 / I don't feel this way about this person<br>3 / I don't know	Alter attributes - ease

Name	Type	Used In
Freq	ordinal<br><br>VALUE / LABEL<br>6 — At least daily<br>5 — Most days of the week<br>4 — At least once a week<br>3 — About every two weeks<br>2 — A few times a month<br>1 — Less than once a month	Per alter form
Knows	layout	Closeness and alter-alter ties<br>Closeness and alter-alter ties
Live_close	ordinal	Per alter form

<table>
<tr><th>Name</th><th>Type</th><th>Used In</th></tr>
<tr><td></td><td>
<table>
<tr><th>VALUE</th><th>LABEL</th></tr>
<tr><td>1</td><td>We live together</td></tr>
<tr><td>2</td><td>They are within walking distance</td></tr>
<tr><td>3</td><td>In the same city, town, or village, but not walking distance</td></tr>
<tr><td>4</td><td>Less than an hour away</td></tr>
<tr><td>5</td><td>More than an hour away but still in Scotland</td></tr>
<tr><td>6</td><td>They live outside of Scotland</td></tr>
<tr><td>7</td><td>They don’t live in the UK</td></tr>
<tr><td>8</td><td>I don't know where they live</td></tr>
</table>
</td><td></td></tr>
<tr><td>Name</td><td>text</td><td>Name generator 1
Name generator 2
Name generator 3
Name generator 4</td></tr>
<tr><td>Postcode</td><td>text</td><td>Per alter form</td></tr>
<tr><td>Rel_length</td><td>ordinal
<table>
<tr><th>VALUE</th><th>LABEL</th></tr>
<tr><td>1</td><td>Less than a year</td></tr>
<tr><td>2</td><td>1-2 years</td></tr>
<tr><td>3</td><td>3-5 years</td></tr>
<tr><td>4</td><td>6-10 years</td></tr>
<tr><td>5</td><td>More than 10 years</td></tr>
</table>
</td><td>Alter attributes - relationship length</td></tr>
</table>

Name	Type	Used In
Relat	categorical<br><br>VALUE / LABEL:<br>1 — Child (including step-children and foster children)<br>2 — Partner/Spouse<br>3 — Parent<br>4 — Other family<br>5 — Friend<br>6 — Colleague<br>7 — Neighbour<br>8 — Other	Alter attributes - relationship to ego

Group

Name	Type	Used In
Group	boolean	Name generator 5 - Groups
Group_age	ordinal<br><br>VALUE / LABEL<br>1 — younger<br>2 — same age<br>3 — older than me<br>4 — a mix of all ages	Group attributes
Group_gender	ordinal<br><br>VALUE / LABEL<br>1 — same gender as me<br>2 — different gender to me<br>3 — mixed gender	Group attributes
Group_location	text	Group attributes
Group_size	number	Group attributes
Group_type	text	Group attributes
Name	text	Name generator 5 - Groups

Place

Name	Type	Used In
Active_there	ordinal<br><br>VALUE / LABEL<br>1 Yes<br>2 No	Place attributes
Affect_activity	ordinal<br><br>VALUE / LABEL<br>1 Very negatively<br>2 A little negatively<br>3 It wouldn't matter<br>4 Positively<br>5 Very positively	Place attributes
Affect_social	ordinal<br><br>VALUE / LABEL<br>1 Very negatively<br>2 A little negatively<br>3 It wouldn't matter<br>4 Positively<br>5 Very positively	Place attributes
Easy_get_there	ordinal<br><br>VALUE / LABEL<br>1 Very easy<br>2 Easy<br>3 Moderate<br>4 Difficult<br>5 Very difficult	Place attributes

Name	Type	Used In
Get_there	ordinal<br><br>VALUE / LABEL<br>1 Walk<br>2 Drive<br>3 Transport<br>4 Get a lift<br>5 Other	Place attributes
Meet_there	ordinal<br><br>VALUE / LABEL<br>1 Yes<br>2 No	Place attributes
Name	text	Name generator - Places

Know_each_other

Name	Type	Used In

No variables to display.

Activities_with

Name	Type	Used In

No variables to display.

Resource Library

Image

NAME	63127038-62273800-bf7f-11e9-914f-bd1c431c76f2.png
TYPE	Image
PREVIEW	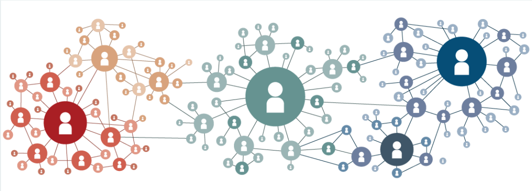

NAME	Debrief.png
TYPE	Image
PREVIEW	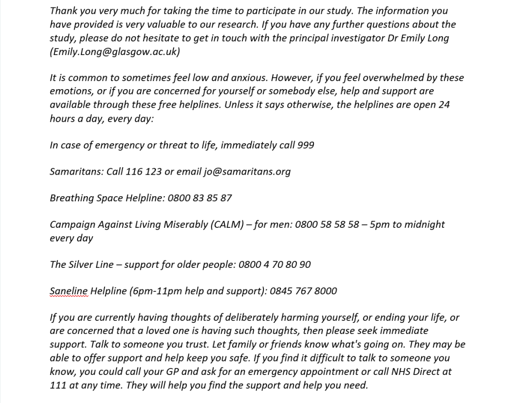

Thank you very much for taking the time to participate in our study. The information you have provided is very valuable to our research. If you have any further questions about the study, please do not hesitate to get in touch with the principal investigator Dr Emily Long (Emily.Long@glasgow.ac.uk)

It is common to sometimes feel low and anxious. However, if you feel overwhelmed by these emotions, or if you are concerned for yourself or somebody else, help and support are available through these free helplines. Unless it says otherwise, the helplines are open 24 hours a day, every day:

In case of emergency or threat to life, immediately call 999

Samaritans: Call 116 123 or email jo@samaritans.org

Breathing Space Helpline: 0800 83 85 87

Campaign Against Living Miserably (CALM) – for men: 0800 58 58 58 – 5pm to midnight every day

The Silver Line – support for older people: 0800 4 70 80 90

Saneline Helpline (6pm-11pm help and support): 0845 767 8000

If you are currently having thoughts of deliberately harming yourself, or ending your life, or are concerned that a loved one is having such thoughts, then please seek immediate support. Talk to someone you trust. Let family or friends know what's going on. They may be able to offer support and help keep you safe. If you find it difficult to talk to someone you know, you could call your GP and ask for an emergency appointment or call NHS Direct at 111 at any time. They will help you find the support and help you need.

Network

NAME	Places V9.csv
TYPE	Network
VARIABLES	name
