# Supplementary material for: Social network characteristics associated with mid-to-older aged adults’ co-engagement in physical activity
Source: PLoS One. 2025 May 7;20(5):e0319981. doi: 10.1371/journal.pone.0319981 (PMC12057854; doi:10.1371/journal.pone.0319981)
Supplement: S2 Appendix — (DOCX) [file pone.0319981.s002.docx]

Appendix 2. Bivariate Correlations of Key Variables
