# Supplementary material for: Social network characteristics associated with mid-to-older aged adults’ co-engagement in physical activity
Source: PLoS One. 2025 May 7;20(5):e0319981. doi: 10.1371/journal.pone.0319981 (PMC12057854; doi:10.1371/journal.pone.0319981)
Supplement: Attachment — Submitted filename: Response to Reviewers.docx [file pone.0319981.s004.docx]

**Manuscript: PONE-D-24-30751**

**Response to reviewers**

Dear Dr Vacchiano,

The authors thank you for the opportunity to submit a revised version of our manuscript, “Social network factors associated with mid-to-older aged adults’ co-engagement in physical activity” for publication in PLOS One. We appreciate your careful consideration and insightful feedback, which we have incorporated to make valuable improvements to the manuscript. These changes are retained as tracked changes within the manuscript. Please see below for our responses to each reviewer comment. All page numbers refer to the revised manuscript with tracked changes.

**Responses to reviewer #1**

Reviewer #1

**Comment:** “The paper adequately justifies the importance of studying older adults. It also recalls the importance of physical activity as a protective factor against morbidity and mortality. On the contrary, sedentary lifestyle becomes an important risk factor, especially for older people. In this context, it aims to explore what type of social contacts facilitate co-engagement in physical activities.”

Author Response: Thank you for your clear summary of our background and aims.

Reviewer #1

**Comment**: “Although it refers to older people, the average age seems relatively low (66 years, which in some countries is close to the average retirement age). Although a decrease in physical activity is observed from the age of 55 onwards, the older population may be interesting to analyse precisely because of the impact it may have on their health.

**Author Response**: We agree that the average age of our sample is relatively low. It is around the middle of our inclusion range of 55-75. We have added some detail to the manuscript, 1) acknowledging that our sample had generally not reached older ages where the health impacts would have fully developed, and 2) that we were specifically interested in the protective effects of co-engagement, therefore there is a value in measuring co-engagement characteristics for people still in their 50s and 60s, as interventions at this stage could prevent health issues as people become older. Prevention is a key element within the World Health Organization (WHO) approach to healthy ageing (Rudnicka et al., 2022).

The revised text reads as follows on page 8:

[“Although our average participant had not generally reached older ages where the age-related health outcomes related to physical activity levels would have fully manifested, we were interested in the preventative effects of co-engagement at an earlier stage, to inform the development of timely interventions”]

**Comment**: The fieldwork lasted more than a year. This is a time in which significant changes in the environment can occur that affect mobility. It is worth remembering the ups and downs in social and physical activity linked to the COVID-19 pandemic, for example.

**Author Response**: We agree that the one-year fieldwork period could capture contextual variation in social and physical activity between participants. In Scotland, the COVID-19 measures that may affect mobility, such as travel restrictions, face covering regulations, and mass testing, had ended before our fieldwork commenced. However, we accept the general point that participants interviewed at different times may have been affected by different environmental factors. Since our primary outcome was co-engagement in physical activity - which included examples, such as going for walk, gardening, playing sports, and exercise classes – rather than individual levels of activity, we would not expect that these variations would have a large impact on our findings. That is, even if individual physical activity levels were affected by temporal context, the tendency to be active with others rather than alone, when active, would remain consistent. This is validated by the fact that we controlled for individual physical activity levels and found these were not significantly associated with co-engagement in our full model. That said, we do find your comment insightful and believe it is worth acknowledging this point in the manuscript. We have added a sentence to the methods section and the limitations section acknowledging the potential for variation, noting that future research could model a wider range of contextual factors.

The revised text reads as follows on page 9:

[“We note that this fieldwork period may have meant that participants were exposed to differences in temporal context that affected their physical activity, such as different weather or policy developments”]

Further revised text reads as follows on page 30:

[“Furthermore, participants were interviewed over a one-year period in which their physical activity levels may have been affected by variation in environmental factors, although we did control for physical activity levels and found no significant effect on co-engagement in our final model”]

Reviewer #1

**Comment**: “The average of 26 contacts is valuable, considering that a large part of the social support literature has focused on fewer than 10 contacts. This affects the structure of the personal network evaluated, as it is a dense core, which usually varies less between respondents. See for example: McCarty, C. (2002). Structure in personal networks. Journal of social structure, 3(1), 20. / Maya Jariego, I. (2018). Why name generators with a fixed number of alters may be a pragmatic option for personal network analysis. American journal of community psychology, 62(1-2), 233-238. / McCarty, C., Lubbers, M. J., Vacca, R., & Molina, J. L. (2019). Conducting personal network research: A practical guide. Guilford Publications. The application of multilevel analysis is also appropriate given the nature of the data”

**Author Response**: Thank you for commenting on the value of capturing larger numbers of social contacts. We agree that allowing participants to name a greater number of social contacts allowed us to capture more variation in social contact and relationship attributes, which is important to fully understand who people co-engage in physical activity with. If limited to, for example, ten contacts, people may be more likely to only named their closer relationships who first come to mind. Our analysis showed that participants often co-engaged with more peripheral relationships, who may not have been captured by a fixed number of alters. We have added the Jariego (2018) citation, which discusses the tendency for fixed alters to homogenise network structures.

The revised text reads as follows on page 9:

[“We opted not to limit the number of social contacts participants could name, in order to capture an appropriate level of variation in who they interact with, allowing the inclusion of weaker social ties that may be overlooked if prompting for a fixed number of contacts (26)”]

Reviewer #1

**Comment**: “There appears to be some overlap between the independent variables referring to relationships and the dependent variable (defined as "co-engagement"). The dependent variable refers not only to the performance of physical activities, but to the performance of physical activities jointly with some members of the network. Relationships are present in the predictor and the outcome. How is participation in physical activities (with other people) separated from the quality, quantity and structure of relationships with them?”

**Author Response**: We have made sure that there is no overlap between the independent variables and dependent variable. These capture conceptually distinct relationship attributes. The model itself separates these variables by calculating the effect of each independent variable on the outcome, controlling for all other predictors, and accounting for random variability between participants. Furthermore, we conducted bivariate analysis of correlations between all variables, including our outcome of co-engagement (Appendix 1). There was only weak correlation between the outcome and any of the relationship predictors included in the models, confirming these as distinct constructs (the most strongly correlated was frequency of interaction, with a weak coefficient of only 0.27). We recognise that readers of the article may also query this, and it would therefore be beneficial to provide some of this explanation within the article.

The revised text reads as follows on page 10:

[“As such, conceptually distinct relationship attributes were used as both predictors and outcome, with the aim of assessing which types of relationship tend to make up the subset our participants were physically active with”]

Further revised text reads as follows on page 17:

[“Bivariate analyses using Pearson’s correlation coefficient confirmed only weak correlations between any combination of variables included in the models, including those measuring separate attributes of relationships, confirming that our outcome was a distinct construct from other relationship attributes used as predictors”]

Reviewer #1

**Comment**: “On the other hand, selection and influence processes can be considered. Personal relationships facilitate physical activity, just as physical activity can facilitate the development of relationships. Why are the direction of relationships chosen over physical activity in relational contexts? Is the alternative explanation corresponding to the other direction of the relationship controlled in any way?”

**Author Response**: Our data collection procedures (described on p.9) involved first asking participants to list their relationships, and then tell us which of these they were active with, identifying a subset of their full network as co-engagement ties. This informs the direction of our analysis. The reviewer is correct that an association between physical activity and relationships may involve both selection and influence processes. As we have conducted a cross-sectional study it would not be possible for our analysis to untangle selection and influence processes. As such, we have been careful to talk in terms of associations between predictors and outcomes, rather than implying a causation. The reason we have chosen co-engagement (dual social/physical activity) as the outcome, is that we believe this outcome has particular relevance for the study population and adds to the literature where most studies use physical activity alone as the outcome. As we have stated in our introduction, “the dual physical and social benefits of co-engagement are particularly important as people progress from mid-to-older age”, because both physical activity and social activity buffer the negative effects of ageing. So, in our study we do not investigate whether relationships influence physical activity, or physical activity influences relationships, but the fact that these occur together with specific contacts, and it is useful to know who these contacts are. This is a similar approach to other network studies that use a specific type of relationship as the outcome (social support, financial support, etc) – we chose co-engagement because we are interested in a specific type of social connection that involves being active. We are interested in these relationships because we want to know the types of people and relationships it may be helpful to include in interventions. Although it would be technically possible to use co-engagement as a predictor, the relationship variables such as relationship quality, length of time known, how close people live, and so on, do not represent our substantive outcome of interest. We have made this clearer in our ‘variables’ section We have also added the investigation of influence and selection processes as a direction for future research in the discussion.

The revised text reads as follows on page 12-13:

[“We selected co-engagement as our outcome variable as it captures a specific type of relationship which the literature suggests is important due to providing dual physical and social benefits (23). Key outcomes of interest for the PACES study were physical activity and social connectedness. The outcome measure represents a point where these intersect and may represent useful levers for interventions (18). This provides a new angle for thinking about physical activity, which is often measured as an individual attribute, rather than something embedded within relationships (10)”]

Further revised text reads as follows on page 30:

[“social selection and influence processes,”]

Reviewer #1

**Comment**: “In that regard, the following conclusion seems confusing: "The insignificance of network size suggests that co-engagement may be driven by a different set of factors than individual physical activity, since network size has been consistently associated with individual activity levels in previous research".

**Author Response**: What we meant here is that previous studies have consistently associated having more social contacts with having higher physical activity levels. We may then hypothesise that people with more social contacts are more likely to co-engage in physical activity with more people. This would make sense, as there would be a greater number of options for people to co-engage with. In our study, having a larger network did not increase the probability of co-engaging in physical activity with contacts. Simply living closer to social contacts and having frequent interaction was more important. Therefore, while network size may affect whether an individual has low, moderate, or high activity levels overall, they don’t necessarily need a large network to be active with others, which has dual physical/social benefits. Hence, we assert that our understanding of factors known to be associated with individual activity cannot be readily applied to our novel study of co-engagement. It is not about the number of relationships, or even the quality of these relationships, it is about their convenience and accessibility (for this age group specifically). We also note that our use of the word ‘driven’ implies that co-engagement is caused by the relationship predictors in the model, when of course it is a simple association.

We accept that the wording of our original statement could be amended and expanded to better communicate this point.

The revised text reads as follows on page 23-24:

[“Network size has been consistently associated with levels of individual physical activity in previous research (10). However, in our study, network size was not significantly associated the probability of co-engagement in physical activity. That is, having a greater number of people to potentially be active with, did not lead to being active with a greater number of people. This suggests that co-engagement, as a phenomenon involving combined physical activity and social interaction, may be associated with different factors than those associated with variation in overall activity levels of individuals.”]

Reviewer #1

**Comment**: “The Methodology is explained in detail and clearly. However, there are some inconsistencies: In the Methodology section, it is stated that the mean age was 66 and, in the Results section, it is stated that it was 65.6. What was the real mean? On the other hand, the sample is clearly biased towards women (72 percent). Does this indicate a problem of accessibility or a refusal to participate by the male population? How does this affect the validity of the results?”

**Author Response**: Thank you to the reviewer for catching this inconsistency in reporting of the mean participant age. The true mean is 65.6, we had rounded this to the nearest whole number in the results section but reported to one decimal place in the findings section. For consistency, we have amended it to read 65.6 in both sections.

The revised text reads as follows on page 8:

[“mean age = 65.6”]

On the second point, we accept the sample is biased towards women, who were more likely to volunteer to participate in the research (not due to refusal of men to participate, but more women proactively volunteering). This pattern has been observed in physical activity research studies more broadly (e.g., Cooke and Jones, 2017). We had not acknowledged this gender bias in our discussion and are glad to do so.

Further revised text reads as follows on page 30:

[“There was also a gender bias, with women overrepresented in our sample. This reflects a broader trend observed in studies relating to physical activity. The Scottish Health Survey (9) records that a lower proportion of women than men meet recommended physical activity levels, in the 55-64, 65-74, and over 70 age groups. However, our analysis controlled for gender of participants and contacts, with no significant effect”]

Reviewer #1

**Comment**: “It is interesting to note that relationships were not particularly relevant predictors of co-engagement. Could it be that the two variables are so closely related that the first does not serve to predict variability in the second? Also interesting is the predictive power of walking the dog, which is mentioned as an added circumstance in the Methodology section.”

**Author Response**: On the first point, we would assert that specific relationship attributes were in fact very relevant predictors of co-engagement, particularly the frequency of interaction, relationship length, and geographic proximity of the relationship. However, it is certainly interesting that relationship quality was not a relevant predictor, as one may theorise that people would be more likely to co-engage with better quality relationships. We have addressed this in the discussion, by framing it within the social convoy model – essentially, variation in relationship quality can be expected, as people in this age group will co-engage with newer contacts who they don’t necessarily have close or supportive relationships with (e.g., if they belong to a walking group, there might be a mix of closer friends and less close acquaintances).

There is no issue with any two variables being too closely related – bivariate analyses did not show high correlations between any of the predictors and the outcome (see Appendix 1. Bivariate Correlations of Key Variables). Any correlation between variables was checked in advance and eliminated from analysis.

On dog walking – we also agree this is interesting and we could have potentially said more about it in the discussion. However, as this was mainly a control variable, rather than a hypothesis testing variable, and was not significant in the final model, we have tried to mainly limit discussion to factors that retained significance in the final model.

**Responses to reviewer #2**

Reviewer #2

**Comment:** “Thank you for the opportunity to review this study. This research aims to understand how co-engagement in physical activity may be related to the social networks of participants. It uses a multilevel approach with ego-alter tie at level 1 and ego characteristics (including the egonet properties) at level 2. This allows to go further than merely considering physical activity as an individual process, by including its interdependence with the persons present in the social surroundings of the individuals. The paper is well structured and the statistical analyses are well handled and performed, with the appropriate design to respond to the research questions. The results are well discussed and implications are also striking. However, a few complementary explanations and issues need to be addressed.”

**Author Response**: Thank you for your positive comments on our manuscript. We are glad for the opportunity to address your comments and resolve any issues raised.”

Reviewer #2

“First, there lacks a precise description of the sample, in particular on how the different social domains (friends, family, colleagues, etc.) are distributed. Without such information, it is hard to tell if the subgroups' sample size are large enough to ensure sufficient statistical power.”

**Author Response**: We thank the reviewer for catching this oversight in our description of the sample. We did check how these social domains were distributed before running analysis but had not reported this in the sample description. We had considered consolidating categories (e.g., family/partner/child vs friend/neighbour/colleague) but felt this would result in unnecessary loss of information. We also note that we found statistically significant results for some of our smaller categories, such as partners, neighbours, and colleagues, suggesting that there was sufficient power in our categories. For clarity, the counts and proportions of relationship types were:

- Friend (n=1671, 45.4%)
- Family (n=877, 23.8%)
- Other (n=393, 10.7%)
- Child (n=250, 6.8%)
- Neighbour (n=211, 5.7%)
- Colleague (n=154, 4.2%)
- Partner (n = 95, 2.6%)
- Parent (n=26, 0.7%)
- NA (n=2, 0.01%)

Due to our large level 1 sample size, and ability to avoid aggregating these observations, we are confident that we have sufficient statistical power to assess differences between relationship subgroups. Note: we have also added more detail about the physical activity levels of our participants to give a more precise description.

The revised text reads as follows on page 11 (Table 1):

[*Additional row added to table summarising distribution of relationship types*]

[*Additional detail added to table summarising distribution of physical activity levels*]

Reviewer #2

“I see that Physical activity is differentially operationalized whether it is the outcome variable or it is the physical activity of the respondent. The outcome variable is adapted from Mötteli and Dohle (2020) and the activity of the respondent is measured with the international IPAQ scale. Although one refers to co-engagement and the other refers to individual physical activity, I am wondering why the authors did not use a consistent measure.”

**Author Response**: Our study did include two different measures relating to physical activity. The IPAQ scale measures individual physical activity levels (summarised as low, moderate, or high) and is a level 2 variable, an attribute of the participant themselves. For example, it contains items such as ‘during the last 7 days, on how many days did you do vigorous physical activities like heavy lifting, digging, aerobics, or fast bicycling’. Co-engagement, our outcome variable, is a level 1 variable which describes a form of connection between the participant and each social contact. The multilevel approach allows us to model variation at both levels simultaneously. Technically, it would not be possible to use the IPAQ measurement for social contacts, as it contains detailed questions about activities which participants would not be able to report for all their contacts. It is also not a relational measure. We were interested in co-engagement as an outcome, as recent literature suggests that co-engagement had dual benefits for this age group and particular relevance for social interventions. We have simply controlled for individual PA levels in our level 2 predictor, so we can ensure that higher levels of co-engagement are not simply due to having higher PA levels in general. Since individual PA (IPAQ) was not a significant predictor of co-engagement in the final model, we can conclude that the factors associated with co-engagement are applicable to people with a variety of PA levels. This is a reasonable query, and we are happy to add detail to the manuscript summarising this explanation.

The revised text reads as follows on page 13:

[“Including this as an independent variable allowed us to control for variation in co-engagement that may have been attributable to variation in individual physical activity levels”]

Reviewer #2

“It misses a rationale for the inclusion of key variables in the analyses. It is the case for the E-I indices and the degree of the alters, for instance. Although it is well discussed in the discussion section, elaborating on the importance of such variables in the literature section and providing hypotheses related to the research question would help understand why they are considered in the analyses.”

**Author Response**: Thank you again for noting an oversight in our description of the methods. These measures were carefully selected to address specific conceptual issues, which have been discussed in the discussion but not covered in the description of the variables. We agree that it is important to elaborate on why we included these variables.

The revised text reads as follows on page 15:

[“Highly connected social contacts may be able to diffuse health behaviours more effectively, as they tend to bridge groups and are interacted with in a greater variety of social contexts (42)”]

Further revised text reads as follows on page 16:

[“This assessment of whether co-engagement contacts tend to have ties to each other was sought to understand whether co-engagement tends to occur within specific pairs of individuals or broader groups, providing insight into the tendency for homophily and the types of intervention development that may be informed (21).”]

Reviewer #2

“The ‘Quality of the relation’ variable is a composite variable, which reaches a good internal consistency of .81. However, due to the different scales and ranges of the three variables (one variable uses a 5-point Likert scale and the two others are binary) some standardization seems to be needed before computing the composite score. Was this considered and can the authors provide a robustness check to see how this would impact this variable?”

**Author Response**: The reviewer is correct that the relationship quality measure is a composite variable which combines measures of different scale. We are glad to confirm that this was considered, and our reporting refers to the standardised alpha, rather than the raw alpha. Therefore, we believe the only amendment to the manuscript is to clarify that we used the standardised alpha for this measure.

For information, we used the ‘alpha’ command from the ‘psych’ package in R, which provides both a raw and standardised alpha. The raw alpha was only 0.67. We also observed that the reliability of the composite scale would have been reduced if any of the three items were dropped, to a raw and standardised alpha of 0.79 if closeness was not included, and to standardised alpha of 0.71 if either ‘count on’ or ‘ease with’ were not included. We are therefore confident in the reliability of our composite measure in its current form.

The revised text reads as follows on page 15:

[(“Cronbach’s standardised alpha: 0.81”)]

Reviewer #2

“Density is included in the 'measure of social network' section but does not seem to be included in the models. Is there a reason for this? Is density only used to compute the E-I indices? Please explicit it as it is unclear.”

**Author Response**:

Density was summarised in an early analysis plan, but preliminary models showed high correlation with other measures, such as degree centrality, and a very wide confidence interval. Given that density did not contribute substantial theory to our paper, and based on these model fit concerns, we elected to remove the variable.

Thank you for catching that density was inadvertently left in our methods section. We have now removed the sentence on p.14 which had stated “*Network density* was a measure of the total proportion of social contacts who were indicated as knowing each other”.

Reviewer #2

“From how the name generator procedure is described, it seems that participants are free to choose the number of alters they elicit. If that is the case, it results in unequal ego network sizes. This can have a potential impact on different variables included in the models as they are dependent on the size of the network. That is the case for degree centrality, as alters have a higher probability of having a higher degree in larger networks. The E-I index is also more likely to be smaller in larger networks. Although size can be considered as a control, are there any other ways the authors took it into account, and were there robustness checks with standardized measures for instance, or by hierarchically including the different variables?”

**Author Response**: The reviewer is correct that we placed no limits on the numbers of social contacts a participant could name. We note that reviewer 1 has also commented on this as a strength of the method, as it captures greater useful variation in social contacts. The reviewer is correct, though, that other measures such as degree and EI index are somewhat dependent on network size. We managed variation in network size by removing outliers below or above 1.5 times the interquartile range and standardising the network size by rescaling to a range of 0 (min value) to 1 (max value). As the reviewer states, we included network size in the models to control for whether differences in EI index were attributable to variation in network size. In our unadjusted model of network structures, we found a negative EI for co-engagement ties significantly predicted the probability of co-engagement, controlling for network size, gender EI, and age EI.

Revised text reads as follows on page 16:

[“Outliers below or above a threshold of 1.5 times the interquartile range were removed, to manage any variation introduced by very large networks, and the measure was standardised by rescaling values within a range of 0 to 1”]

Regarding robustness checks, we did hierarchically include variables in our initial modelling, starting with network size then incrementally adding the EI indices. The results showed that co-engagement EI index had a significant effect after controlling for network size and gender/age EI. However, we had not standardised the EI measures onto the same 0 to 1 scale as the network size, believing that the small -1 to 1 range of EI index was sufficiently scaled. This range was also valuable for interpretation, as positive and negative ties indicate external/internal direction. As a robustness check, we have rerun the model with all EI indices rescaled accordingly. The results remain consistent, with EI for physical activity having a significant effect when controlling for the other measures, and none of these other measures having a significant effect. We have also rerun the models with degree standardised to a range of 0 to 1 and found results are consistent.

Revised text reads as follows on page 18:

[“Robustness checks were carried out using standardised measures of social contact degree and EI indices, with the direction and significance of all predictors found to remain constant in adjusted and unadjusted models”]

**Responses to reviewer #1 and #2**

Please note that we have made some minor changes for clarity and presentation when redrafting the manuscript.

- We have added a footnote to the results table that our reference category for relationship types is ‘partner’.
- Replaced ‘ego’ and ‘alter’ language from table 2 in favour of ‘participants’ and ‘social contacts’, in keeping with the rest of the manuscript.
- Changed ‘female’ to ‘woman’ in table 1 consistent with rest of manuscript.
- Changed ‘detailed’ to ‘novel’ on p.23.
- Removed one author who did not feel they had contributed substantially.
- Formatted according to PLOS One formatting guidelines.
- Added ethics statement to methods section on p.9.
- Checked for typos and clarity throughout.
