# Supplementary material for: Social network characteristics associated with mid-to-older aged adults’ co-engagement in physical activity
Source: PLoS One. 2025 May 7;20(5):e0319981. doi: 10.1371/journal.pone.0319981 (PMC12057854; doi:10.1371/journal.pone.0319981)
Supplement: Attachment — Submitted filename: Response_to_Reviewers_auresp_2.docx [file pone.0319981.s005.docx]

**Manuscript: PONE-D-24-30751R1**

**Response to reviewers**

Dear Dr Vacchiano,

The authors thank you for the opportunity to submit minor revisions to our manuscript, “Social network factors associated with mid-to-older aged adults’ co-engagement in physical activity” for publication in PLOS One. We are grateful for the positive feedback on our revised manuscript from the original reviewer #2. Thank you to reviewer #3 for agreeing to review this revised manuscript. Please see below for our responses to the additional points raised by reviewer #3. All page numbers refer to the revised manuscript with tracked changes.

**Responses to reviewer #3**

Reviewer #3

**Comment:** “(1) Your results show that new relationships, particularly those that are not close, and the geographical proximity of alters with whom participants engage in physical activity are predictors of co-engagement. I was wondering if social participation, in terms of belonging to associations, clubs, etc., might be a hidden variable explaining co-engagement in physical activity. Clubs or associations tend to be local, bringing together people who live nearby but are not closely related. Additionally, they structure participation based on activity schedules and group people with similar characteristics (e.g., retired older adults, same age). Have you considered including membership in associations (e.g., walking clubs, sports clubs, etc.) in your research and model?”

Author Response: This is an insightful point. Certainly, participant involvement in clubs and associations could explain tendencies to co-engage with newer relationships, less close relationships, and people who live nearby. However, capturing data specific to group membership, linked to network ties, was deemed prohibitively time-intensive and burdensome to participants in the present study. As such, we did not capture group membership at the level of social contacts (level-1). For example, we did not have a name interpreter to capture information on which social contacts were interacted with in community groups. For this reason, we cannot untangle or control for whether participants were more likely to co-engage with social contacts they attended groups with. That is, we are unable to test whether the tendency to co-engage with social contacts in the same age, who live closer, applies mainly to people they see at groups.

We recognise that an observant reader may have the same query and are happy to note, as a limitation and direction for future research, that the role of groups, association, clubs etc could be an important area to be explored. The amended text reads as follows on p.29

[“Finally, our analysis did not model whether participants or their social contacts belonged to groups, clubs, or met at certain geographic settings.”]

Further amended text reads as follows on p.29

[“the role of community groups and clubs in shaping social network characteristics”]

*Note: As a further response to the reviewer, we are exploring the role of group membership in parallel workstreams in the PACES project – though, not connected to the present paper. Within this wider work, we use longitudinal qualitative methods and participatory systems mapping workshops to explore how groups may facilitate social connection. We look forward to sharing these results in future publications.

**Comment:** “(2) In your paper, you have identified the types of social contacts positively associated with co-engagement. Conversely, are there any that are negatively associated with co-engagement? Are there certain types of social contacts that tend to hinder co-engagement in physical activity?

Author Response: Our findings did show some negative association, where specific relationship types were associated with lower odds of co-engagement. One of these was relationship length, where knowing a person longer decreased the odds of co-engagement (reported on p.20 and discussed p.24-26). Other negative associations significant in the final adjusted model were ‘neighbour’ (OR = 0.11, 95% CI = 0.04, 0.28), ‘family member’ (OR = 0.27, 95% CI = 0.14, 0.53), and ‘colleague’ (OR = 0.06, 95% CI = 0.01, 0.22). Note that as there were separate categories for partner, child, parents etc, that ‘family’ would apply to family members not in these categories.

We had commented on this in the discussion on p.26, “There is less of a tendency for intergenerational co-activity or for mid-to-older aged adults to be co-active with neighbours or work colleagues. Following intervention theory, we argue that interventions are more likely to succeed if they capitalise on the naturally occurring tendency to co-engage with partners and friends (of varying closeness) on p.26: “… interventions may have an increased chance of success if they aim to facilitate and enhance activity between people with this naturally occurring tendency to co-engage in physical activity (21)”

**Comment:** “(3) Regarding types of relationships in networks, 11% are classified as "others". Do you have any additional information about this category? Who are they?”

Author Response: In the survey, there was no free-text option for participants who selected ‘other’ here. However, we feel it is worth noting in our variables section on p.14 when describing the relationship type values.

The amended text reads as follows on p.14:

[There was no free-text option to capture additional information on relationships described as other.]

*Note: our parallel workstreams using qualitative interviews and participatory systems thinking workshops will be able to explore important relationship types that were not captured in this social network survey. These findings will be integrated via pillar integration methods and published separately.

**Comment:** “(4) In the "co-engagement in physical activity" section, you wrote: "Participants could assign previously nominated social contacts to this category or add new ones." Does this mean that with this second name generator based on physical activities, participants could extend their "interaction" network after using the first generator? Could you clarify this point?”

Author Response: Yes, this is a feature available in network canvas when using multiple name generators. It is possible, at each name generator stage, to display a ‘roster’ of names that were provided in previous name generator stages. This appears along with the option to add additional names not in the roster. Therefore, participants could indicate which of the social contacts previously nominated they were active with or nominate new social contacts they had overlooked in the previous name generators. It avoids duplication of social contacts who meet the criteria for more than one name generator.

We thank the reviewer for pointing out that this could be clearer in the manuscript. In response, we have added more detail to the description of name generators on p.8, clarified the quoted sentence in the ‘co-engagement in physical activity’ section on p.11, and added further detail to the explanation of network size on p.15, that the full network size combines all name generators.

Amended text reads as follows on p.8 (original text in bold):

[“We used multiple name generators to capture the multidimensional nature of participant social contacts and combined nominations into a single overall social network. **Participants were asked to name the people with whom they had interacted in the last month, people they had not interacted with, but still considered themselves connected to, and which of these social contacts they were physically active with (see measures below)**. For contacts they were physically active with, there was also an option to select from a roster of names generated by the first two prompts.

Further amended text reads as follows on p.11

[“Participants could assign previously nominated social contacts to this category or add additional social contacts that they recalled in response to this specific prompt, with all nominated social contacts combined as their social network and an additional binary variable indicating which of these they were active with.”

Further amended text reads as follows on p.15 (original text in bold):

[“***Network size* was calculated as the total number of social contacts that the participant mentioned**, across all name generators.”

**Comment:** “(5) The degree of each social contact was calculated based on the connection "know each other" or "do physical activity with." This is not entirely clear to me.”

Author Response: This measure was calculated on whether the social contacts know each other. That is, a social contact with six connections to other social contacts would have a degree of six. In the manuscript, we had framed this on p.14 as “The *degree* of each social contact (i.e., the number of connections they have to other social contacts) was calculated as a measure of their centrality within the network”. We have updated the manuscript to be clear it is based on the prompt of whether they know each other. The definition of knowing each other was provided on p.8-9 as “whether they could have a conversation without the participant being present”.

Amended text reads as follows on p.14 (original text in bold):

[“**The *degree* of each social contact (i.e., the number of connections they have to other social contacts**, captured by asking if social contacts knew each other) **was calculated as a measure of their centrality within the network**”]

**Comment:** “(6) In Table 2, significant results are not marked with stars as usual but with confidence intervals in bold. It would be helpful to explain this format in advance for readers who may not be familiar with it. Additionally, it would be beneficial to recall your dependent variable in the first row of your table.”

Author Response: Thank you for this helpful suggestion. We have added a footnote to the table explaining that significant confidence intervals are those in bold.

Amended text reads as follows on p.19:

[“Significant confidence intervals are indicated in bold”]

We have also updated the table title to be clear that table 2 presents predictors of co-engagement in physical activity. We have opted to clarify the title rather than manually adding new rows to the table and have checked that recent Plos One publications also use this format.

Amended text reads as follows on p.18 (original text in bold):

[“**Results of Multilevel Models** Predicting Co-engagement in Physical Activity”]
